# Influence of Light Emitting Diode-Derived Blue Light Overexposure on Mouse Ocular Surface

**DOI:** 10.1371/journal.pone.0161041

**Published:** 2016-08-12

**Authors:** Hyo Seok Lee, Lian Cui, Ying Li, Ji Suk Choi, Joo-Hee Choi, Zhengri Li, Ga Eon Kim, Won Choi, Kyung Chul Yoon

**Affiliations:** 1 Department of Ophthalmology, Chonnam National University Medical School and Hospital, Gwangju, South Korea; 2 Department of Biomedical Sciences and Center for Creative Biomedical Scientists, Chonnam National University, Gwangju, South Korea; 3 College of Veterinary Medicine and BK 21 PLUS Project Team, Chonnam National University, Gwangju, South Korea; 4 Xiamen Eye Center of Xiamen University, Xiamen, Fujian, China; 5 Department of Pathology, Chonnam National University Medical School and Hospital, Gwangju, South Korea; Tokai University, JAPAN

## Abstract

**Purpose:**

To investigate the influence of overexposure to light emitting diode (LED)-derived light with various wavelengths on mouse ocular surface.

**Methods:**

LEDs with various wavelengths were used to irradiate C57BL/6 mice at an energy dose of 50 J/cm^2^, twice a day, for 10 consecutive days. The red, green, and blue groups represented wavelengths of 630 nm, 525 nm, and 410 nm, respectively. The untouched group (UT) was not exposed to LED light and served as the untreated control. Tear volume, tear film break-up time (TBUT), and corneal fluorescein staining scores were measured on days 1, 3, 5, 7, and 10. Levels of interferon (IFN)-γ, interleukin (IL)-1β, IL-6, and tumor necrosis factor (TNF)-α were measured in the cornea and conjunctiva using a multiplex immunobead assay at day 10. Levels of malondialdehyde (MDA) were measured with an enzyme-linked immunosorbent assay. Flow cytometry, 2’7’-dichlorofluorescein diacetate (DCF-DA) assay, histologic analysis, immunohistochemistry with 4-hydroxynonenal, and terminal deoxynucleotidyl transferase-mediated dUTP-nick end labeling (TUNEL) staining were also performed.

**Results:**

TBUT of the blue group showed significant decreases at days 7 and 10, compared with the UT and red groups. Corneal fluorescein staining scores significantly increased in the blue group when compared with UT, red, and green groups at days 5, 7, and 10. A significant increase in the corneal levels of IL-1β and IL-6 was observed in the blue group, compared with the other groups. The blue group showed significantly increased reactive oxygen species production in the DCF-DA assay and increased inflammatory T cells in the flow cytometry. A significantly increased TUNEL positive cells was identified in the blue group.

**Conclusions:**

Overexposure to blue light with short wavelengths can induce oxidative damage and apoptosis to the cornea, which may manifest as increased ocular surface inflammation and resultant dry eye.

## Introduction

A light emitting diode (LED) is a complex semiconductor that emits narrow-spectrum light when a suitable energy is supplied to the leads. It has been developed as an alternative option to replace traditional light sources, and is increasingly used as a lighting component in various electrical appliances, such as televisions, personal computers, and smart phones. From a technical point of view, use of LED as an illumination source is efficient, because it is energy saving, and long lasting compared to pre-existing light sources, such as incandescent light. However, LEDs are known to emit quite a large amount of blue light [1–3].

Humans are constantly exposed to various types of lights that illuminate our surroundings. Light, ranging from x-rays and other ionizing radiation to infrared and longer wavebands, can cause hazardous effects to the eye if it reaches a level capable of causing photochemical reactions, photothermal damage, or metabolic disturbances. Various light-induced ocular pathologies have been recognized, including photokeratitis, pterygium, climatic droplet keratopathy, cataract, and corneal and retinal degeneration [4–7]. Recently, the detrimental effect of blue light on the retina has been extensively investigated [8–10]. Blue light has been known to cause photoreceptor cell and retinal pigment epithelial cell (RPE) damage through excessive reactive oxygen species (ROS) production.

Increased oxidative stress has been documented in mouse models of dry eye and in the conjunctival epithelial cells of patients with dry eye disease [11–15]. However, to our knowledge, few studies have investigated the effects of blue light on the ocular surface, which is directly exposed to light. Niwano et al. [16] showed that blue light in the near-ultraviolet (UV) region may be harmful to *in vitro* mitotic-phase corneal epithelial cells in a dose-dependent manner. In addition, we previously reported that overexposure to blue light *in vitro* can decrease cellular viability and induce significant ROS production compared with other visible light wavelengths from LED [17].

In the present study, we aimed to investigate the effect of LED-derived blue light overexposure on ocular surface health in a mouse model by measuring various clinical and experimental parameters.

## Materials and Methods

This research protocol was approved by the Chonnam National University Medical School Research Institutional Animal Care and Use Committee (CNU IACUC-H-2015-12). All procedures were performed according to the Association for Research in Vision and Ophthalmology statement for the Use of Animals in Ophthalmic and Vision Research. Female C57BL/6 mice aged 6 to 8 weeks were used in the following experiments. The animals were allowed to acclimate for one week before the experiment began. They were housed under standard laboratory conditions with a 12:12 hour light-dark cycle light 8 AM-8 PM; dark 8 PM-8 AM) in the Chonnam National University Hospital animal facilities during the experiment period. The facility temperature was maintained at 25 ± 3°C with 50 ± 5% relative humidity. Food and water were supplied ad libitum.

### LED Light Source and Irradiation

**Fig 1:**
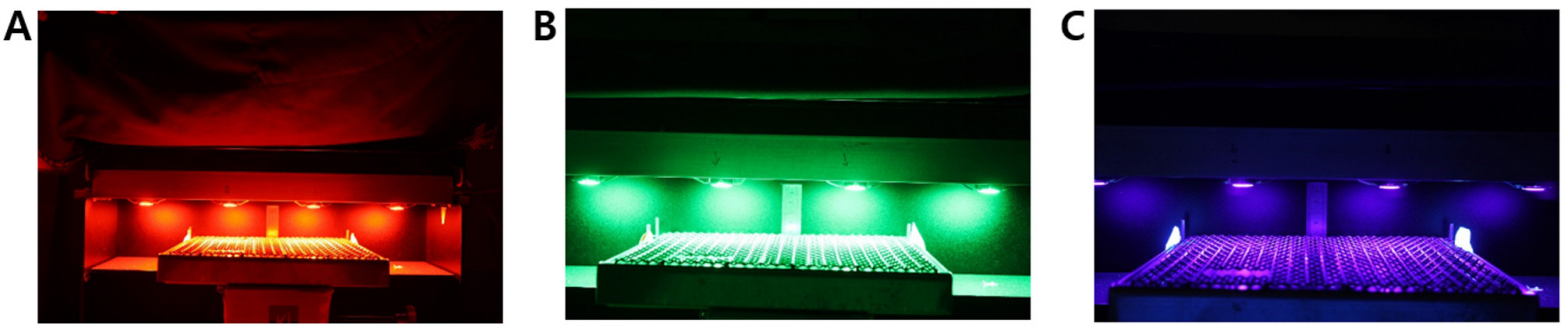
Light emitting diode devices and retaining cages of red light (630 nm) (A), green light (525 nm) (B) and blue light (410 nm) (C).

Three LED lamps with different wavelengths were used. Wavelengths and irradiances are listed in Table 1. The irradiance of each LED was measured with a quantum photoradiometer (Delta OHM, Padova, Italy) connected to a visual probe (Sonda LP 9021 RADl Delta OHM). Animals were separated into four groups, each consisting of six mice. Red, green, and blue group mice were exposed to wavelengths of 630 nm, 525 nm, and 410 nm, respectively (Table 1). The untouched group (UT) was not exposed to the LED light, and served as an untreated control during the experiment. The animal’s ocular surface was exposed to LED-derived light in the following manner. For *in vivo* exposure, mice were confined in an adjustable retaining cage in a dark room, where the LED was placed 5 cm above and perpendicular to the mouse’s head (Fig 1); only the ceiling of the cage emitted light. Therefore, at any given instant, the dose of light on the mouse ocular surface depended upon its head posture. We estimated that the mouse kept its head aligned with its body on average while in the retaining cage. Mice were irradiated with 50 J/cm^2^ twice daily (irradiation began at 9 PM and 4 AM to avoid variation) for a consecutive 10-day period. The irradiance and frequency of radiation were selected based on the data of our preliminary pilot experiments (data not shown). Clinical parameters, including tear volume, tear film break-up time (TBUT), and corneal fluorescein staining scores were measured in that order during the experimental period in four groups (on days 1, 3, 5, 7, and 10). The clinical parameter measurements were taken after two hours after the end of the second irradiation in the standard environment. Animals were kept immobile by intraperitoneal injection of 1 mg pentobarbital during clinical parameters measurements.

**Table 1:** Wavelength and irradiance of light emitting diode (LED) lamps.

LED wavelength (nm)	Irradiance (mW/cm^2^)
630 ± 8	48.8
525 ± 2	59.5
410 ± 10	29.2

Data are expressed as the mean ± standard deviation.

At the end of the experiment, animals were sacrificed with an intraperitoneal overdose of pentobarbital, then multiplex immunobead assay, malondialdehyde (MDA) level measurement with enzyme-linked Immunosorbent Assay (ELISA), flow cytometric analysis, 2’7’-dichlorofluorescein diacetate (DCF-DA) assay, histology, and terminal deoxynucleotidyl transferase-mediated dUTP-nick end labeling (TUNEL) staining were performed after tissue harvesting. All efforts were made to minimize suffering. All experiments and analysis were repeated three times.

### Tear Volume Measurements

Tear volume was measured with phenol red-impregnated cotton threads (Zone-Quick; Oasis, Glendora, CA, USA) as previously described [18]. The lower eyelid was pulled down slightly, and the tips of the threads were placed on the peripheral conjunctiva at the lateral canthus for 20 seconds. The tear volume, expressed in millimeters of thread wet by the tear that turned red, was measured using a microscope (SMZ 1500; Nikon, Melville, NY, USA). Each eye was tested three times, and the average length of red thread was recorded as the definitive value. The measured uptake of tear in millimeters was compared with a standard curve prepared from cotton threads of a known uptake volume of stock basic solution (1,500 mL of 0.9% saline and 5mL of 5M NaOH) over 20 seconds, with volumes in the range that would be expected in mouse tear.

### Evaluation of Tear Film Break-up Time and Corneal Fluorescein Staining

The TBUT and corneal fluorescein staining score measurements were conducted as previously described [19]. One percent sodium fluorescein (1 μL volume) was instilled into the inferior conjunctival sac using a micropipette. After three blinks, TBUT was recorded in seconds using slit lamp biomicroscopy (BQ-900, Haag-Streit, Bern, Switzerland) under cobalt blue light. Ninety seconds later, punctate staining on the corneal surface was evaluated in a masked fashion. Each cornea was divided into four quadrants that were scored individually. The intensity of corneal fluorescein staining was calculated using a 4-point scale; 0, absent; 1, superficial stippling micropunctate staining < 30 spots; 2, punctate staining > 30 spots, but not diffuse; 3, severe diffuse staining but no positive plaque/patch; and 4, positive fluorescein plaque/patch. The scores of the four areas were summed to generate a final grade, ranging from 0 to 16.

### Multiplex Immunobead and Enzyme-linked Immunosorbent Assay

A multiplex immunobead assay (Luminex 200; Luminex Corp., Austin, TX, USA) measured concentrations of interferon (IFN)-γ, interleukin (IL)-1β, IL-6, and tumor necrosis factor (TNF)-α in the cornea and conjunctiva, as previously described [20]. The tissues were collected and pooled in lysis buffer containing protease inhibitors for 30 minutes. Cell extracts were centrifuged at 14,000g for 15 minutes at 4°C, and the supernatants were stored at –70°C until use. Next steps of multiplex immunobead assay were processed within 2 days after storage. Total protein concentration in the supernatants was determined, and 25 μL of total protein of each sample was pipetted into assay plate wells. The supernatants were added to wells containing the appropriate cytokine bead mixture that included mouse monoclonal antibodies specific for the cytokines for 60 min. After three washes, the biotinylated secondary antibody mixture was applied for 30 min in the dark at room temperature. The reactions were detected after addition of streptavidin-phycoerythrin with an analysis system (xPONENT, Austin, TX, USA). The concentrations of the tissue cytokines were calculated from standard curves of known concentrations of recombinant mouse cytokines. Total protein levels of lipid peroxidation markers, MDA, were detected using ELISA. The supernatants were collected and assayed for MDA (Cell Biolabs, San Diego, CA, USA) using the ELISA kit. The samples were analyzed according to the manufacturer’s instructions [15,21]. To find out if storage time influences the results, we additionally performed multiplex immunobead and enzyme-linked immunosorbent assay in 6 normal eyes, right after tissue collection, as control samples.

### Histology

Eyes were surgically excised, washed immediately with ice-cold saline to remove as much blood as possible, fixed in 4% paraformaldehyde, and embedded in paraffin. Next, six-micrometer conjunctival sections were stained with periodic acid-Schiff (PAS) reagent. Sections were examined and photographed with a microscope (BX53; Olympus, Tokyo, Japan) equipped with a digital camera (F2; Foculus, Finning, Germany). Goblet cell density in the superior and inferior conjunctiva was measured in three sections from each eye using image analysis software (Media Cybernetics, Silver Spring MD) and expressed as the number of goblet cells per 100 μm.

### Flow Cytometry

Flow cytometry was performed for quantification of CD4+CCR5+ T cells from the conjunctiva and cornea with a previously described method [22]. The tissues were teased and shaken at 37°C for 60 minutes with 0.5 mg/mL collagenase type D. After grinding with a syringe plunder and passage through a cell strainer, cells were obtained, centrifuged, and resuspended in PBS with 1% bovine serum albumin. After washing, the samples were incubated with fluorescein-conjugated anti-CD4 antibody (BD Biosciences, San Jose, CA), phycoerythrin-conjugated anti-CCR5 antibody (BD Biosciences), and isotype control antibody at 37°C for 30 minutes. The number of CD4+CCR5+ T cells was counted by using a FACSCalibur cytomemter with CellQuest software (BD Biosciences).

### Measurement of cellular ROS production

The level of ROS production was measured using a DCF-DA assay kit according to the manufacturer’s protocol.

DCF-DA is a non-fluorescent, membrane permeable compound that becomes fluorescent and membrane impermeable upon oxidation. Whole-corneal epithelium was scraped with an ophthalmic surgical blade and placed in a 96-well plate containing 200 μL of Krebs-Ringer bicarbonate buffer. The cells were incubated in the dark with 20 μg/mL 2’7’-dichlorofluorescein for 30 minute at 37°C. The plates were read at an excitation of 480 nm and emission of 530 nm (FACSCalibur cytometer; BD Biosciences).

### TUNEL staining

A TUNEL assay was used to detect 3’ hydroxyl ends in fragmented DNA as an early event in the apoptotic cascade and identify apoptotic cells. After tissue preparation described above, staining was performed using the DeadEnd^TM^ Fluorometric TUNEL System (Promega, Madison, WI) according to the manufacturer’s instructions [23,24]. Stained tissues were mounted on slides and the nuclei were visualized with 4′,6-diamidino-2-phenylindole (DAPI) present in the ProLong Gold Antifade Mounting Medium (Invitrogen, Carlsbad, CA) and observed on a Leica TCS SP5 AOBS laser scanning confocal microscope (Leica Microsystems, Heidelberg, Germany) using a Leica 63x (N.A. 1.4) oil objective. Cell images were obtained separately with the following fluorescence excitation and emission settings: excitation at 405 and 488 nm and emission between 424–472 and 502–550 nm for TUNEL assay and DAPI, respectively. TUNEL-positive cells and nuclear staining with DAPI were viewed under a fluorescent microscope.

### Statistical Analysis

SPSS version 18.0 (SPSS, Chicago, IL, USA) was used for statistical analyses. Results are presented as mean ± standard deviation (SD). Normal distribution of the data was verified using the Kolmogorov-Smirnov test. Statistical differences in tear volume, TBUT, and corneal fluorescein staining among the groups was determined by repeated measure analysis of variance (RM-ANOVA) tests followed by Dunnett’s post hoc tests (sphericity assumptions were tested with a Mauchly’s test. In the case of violation, data were adjusted with an Epsilon Greenhouse-Geisser statistic). The Kruskal-Wallis test with Bonferroni post hoc analysis was used to compare cytokines, MDA and 8-OHdG levels, DCF-DA, goblet cell density, and flow cytometry value differences between the groups. A *P*-value < 0.05 (if needed, multiplicity adjustment was used) was considered statistically significant.

## Results

### Tear Volume

**Fig 2:**
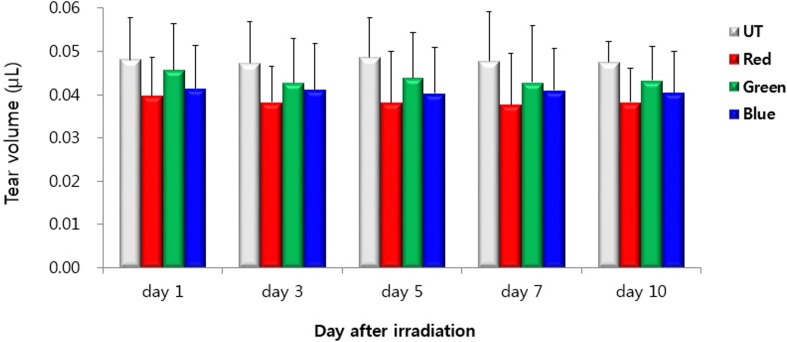
Tear volume in the untouched (UT), red, green, and blue groups at days 1, 3, 5, 7, and 10.

The mean tear volumes in the UT, red, green, and blue groups at day 1 were 0.05 ± 0.01 μL, 0.04 ± 0.01μL, 0.05 ± 0.01 μL, and 0.04 ± 0.01 μL respectively. (Fig 2). The mean tear volumes were 0.05 ± 0.01 μL, 0.04 ± 0.01 μL, 0.04 ± 0.01 μL, and 0.04 ± 0.01 μL at day 3 and day 5 in the UT, red, green and blue groups, respectively. The mean tear volume in the UT, red, green, and blue groups at day 7 and day 10 were 0.05 ± 0.01 μL, 0.04 ± 0.01μL, 0.04 ± 0.01 μL, and 0.04 ± 0.01 μL, respectively. There were no statistically significant differences in tear volumes among the groups during the experiment period (all *P* > 0.05). In addition, the tear volume of each group at days 3, 5, 7, and 10 showed no statistically significant difference when compared with the tear volume of each group at day 1.

### TBUT

**Fig 3:**
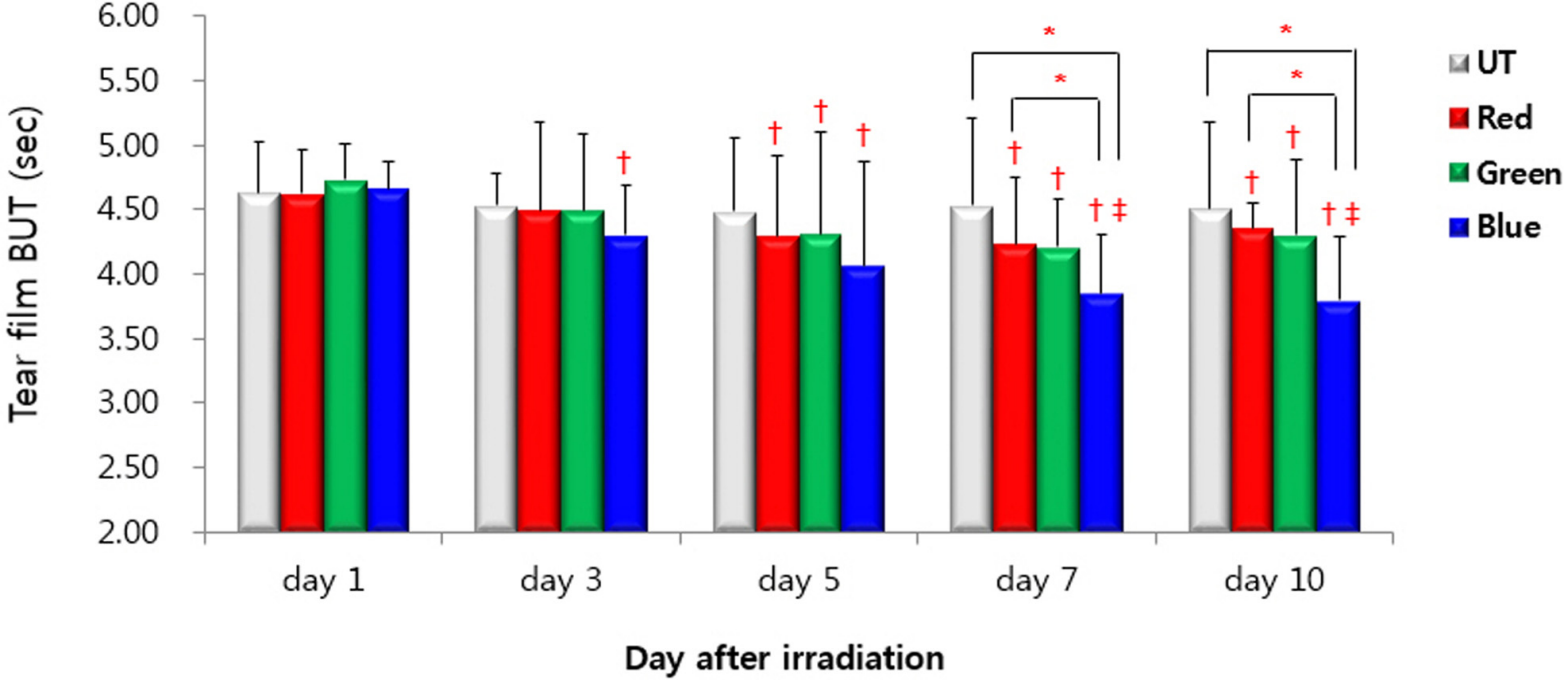
Tear film break-up time in the untouched (UT), red, green, and blue groups at days 1, 3, 5, 7, and 10. * *P* < 0.05 compared between groups. ^†^ *P* < 0.05 compared with the day 1 value. ^‡^ *P* < 0.05 compared with the day 3 value.

TBUTs for the UT, red, green, and blue groups at day 1 were 4.64 ± 0.39 s, 4.62 ± 0.34 s, 4.73 ± 0.28 s, and 4.66 ± 0.22 s, respectively (Fig 3). Mean TBUT in the four different groups showed no statistically significant differences. TBUT of the blue group showed a significant decrease at day 3 of LED irradiation compared with its day 1 value (4.30 ± 0.39 s; *P* < 0.001). At days 5, 7, and 10 of LED irradiation, a significant decrease in TBUT was observed in the red, green, and blue groups compared with day 1 values from the corresponding group (day 5: 4.30 ± 0.62 s, 4.31 ± 0.80 s, 4.07 ± 0.82 s, *P* = 0.017, 0.010, and < 0.001, respectively; day 7: 4.24 ± 0.51 s, 4.21 ± 0.37 s, 3.85 ± 0.46 s, *P* = 0.005, < 0.001, < 0.001, respectively; day 9: 4.36 ± 0.19 s, 4.30 ± 0.59 s, 3.80 ± 0.49 s, all *P* ≤ 0.001) Moreover, in the blue group, TBUTs of days 7 and 10 showed a significant decrease when compared with day 3 values (3.85 ± 0.46 s, 3.80 ± 0.49 s versus 4.30 ± 0.39 s, *P* = 0.012 and 0.001, respectively). As for inter-group comparisons, there were no statistically significant differences in TBUT among the groups at days 1, 3, and 5 (all *P* > 0.05). However, TBUT was significantly shorter in the blue group compared with UT and red groups at days 7 and 10. (day 7: *P* = 0.001 and 0.048, respectively; day 9: *P* = 0.001 and 0.045, respectively).

### Corneal Fluorescein Staining

**Fig 4:**
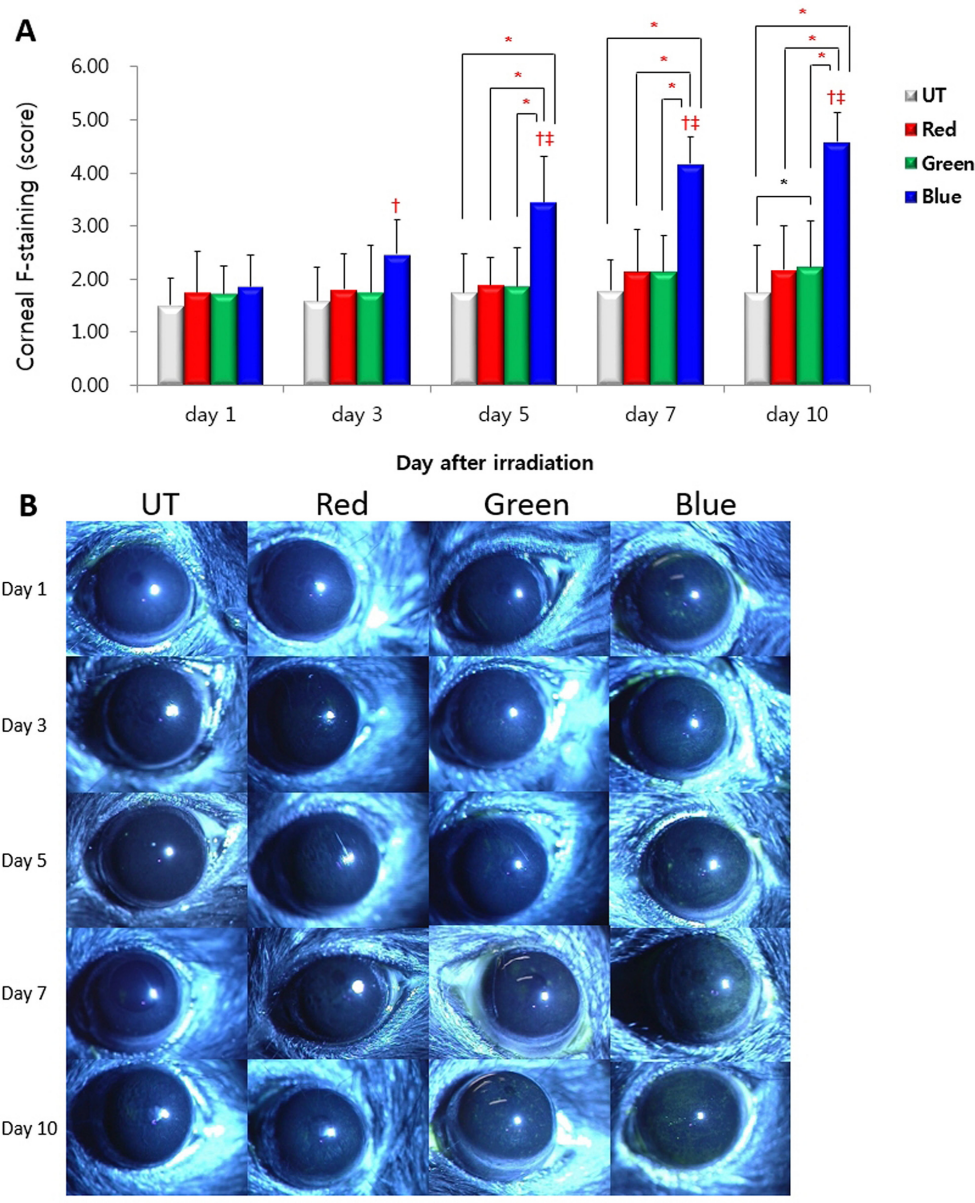
Corneal fluorescein staining scores (A) and representative figures (B) in the untouched (UT), red, green and blue groups at day 1, 3, 5, 7, and 10. * *P* < 0.05 compared between groups. ^†^ *P* < 0.05 compared with the day 1 value. ^‡^ *P* < 0.05 compared with the day 3 value.

Corneal fluorescein staining scores for the UT, red, green, and blue groups at day 1 were 1.50 ± 0.51, 1.75 ± 0.77, 1.72 ± 0.51, and 1.86± 0.59, respectively (Fig 4). The mean staining score showed no statistically significant inter-group differences (all *P* > 0.05). In the blue group, at days 3, 5, 7, and 10, corneal staining scores significantly increased compared to those of day 1 (2.47 ± 0.65, 3.44 ± 0.88, 4.17 ± 0.51, and 4.58 ± 0.55 versus 1.86 ± 0.59, respectively, all *P* ≤ 0.001). Moreover, the blue group showed significantly increased corneal staining scores on days 5, 7, and 10 than on day 3 (all *P* ≤ 0.007). As for inter-group comparisons, irradiation with blue LED led to significant deterioration of corneal staining scores compared to UT, red, and green LEDs at days 5, 7, and 10 (day 5: 3.44 ± 0.87 versus 1.75 ± 0.73, 1.89 ± 0.52, and 1.86 ± 0.72, *P* = 0.028, 0.043 and 0.045, respectively; day 7: 4.17 ± 0.51 versus 1.78 ± 0.59, 2.14 ± 0.80, and 2.14 ± 0.68, *P* = < 0.001, 0.025, and 0.023, respectively; day 10: 4.58 ± 0.55 versus 1.75 ± 0.87, 2.17 ± 0.85, 2.22 ± 0.87, *P* = < 0.001, 0.038, and 0.021, respectively). At day 10, the green group showed an increased corneal staining score compared with the UT group (*P* = 0.043).

### Multiplex Immunobead and Enzyme-linked Immunosorbent Assay

The results of inflammatory cytokines and MDA levels in corneal and conjunctival tissues are shown in Table 2. The concentration of IL-1β and IL-6 in the cornea of the blue group significantly increased 10 days after LED irradiation compared with the UT group (*P* = 0.018 and 0.001, respectively). Moreover, blue LED irradiation significantly increased IL-1β concentration compared with green LED irradiation (*P* = 0.030), and significantly increased IL-6 concentration compared with red and green LED irradiation (*P* = 0.003 and 0.002, respectively). Concentration differences of IL-1β between the red group and blue group did not show statistical significance (*P* = 0.056). No differences in conjunctival inflammatory cytokine levels were observed between groups. As for lipid peroxidation markers, levels of MDA in the blue group cornea significantly increased compared to the UT, red, and green groups (all *P* < 0.01). The blue group also showed significantly elevated conjunctival MDA levels compared to the UT and green groups (*P* = 0.035 and 0.026, respectively). Level of cytokines used in our study and MDA did not show significant differences between normal eyes and UT groups (all *P* > 0.05).

**Table 2:** Concentration of interferon (IFN)-γ, interleukin (IL)-1β, IL-6, tumor necrosis factor (TNF)-α, and malondialdehyde (MDA) in the cornea and conjunctiva of the normal eyes and untouched (UT), red, green, and blue groups.

Tissue	Group	IFN-γ	IL-1β	IL-6	TNF-α	MDA
Cornea (pg/ml)	Normal	10.35±0.25	6.70±0.35	3.25±0.85	1.96±0.18	0.48±0.05
	UT	10.96±0.38	6.69±1.05	3.40±0.92	1.93±0.12	0.51±0.02
	Red	13.22±1.03	7.90±0.39	5.17±0.70	2.12±0.08	0.56±0.03
	Green	12.56±0.57	7.56±0.76	7.05±1.51	2.19±0.04	0.57±0.03
	Blue	15.70±0.48	11.90±1.12*^‡^	20.07±1.47*^†^^‡^	3.59±0.06	2.89±0.06*^†^^‡^
Conjunctiva (pg/ml)	Normal	6.08±0.31	4.35±0.15	11.28±0.86	0.54±0.08	0.75±0.02
	UT	6.02±0.27	4.40±0.10	11.33±1.14	0.56±0.06	0.74±0.02
	Red	6.38±0.06	4.55±0.29	11.47±1.28	0.56±0.07	0.80±0.01
	Green	6.21±0.15	4.61±0.16	11.40±1.33	0.59±0.12	0.79±0.05
	Blue	6.34±0.09	4.82±0.08	12.21±1.39	0.61±0.11	1.22±0.10*^‡^

* *P* < 0.05 compared with the UT group

^†^ *P* < 0.05 compared with the red group

^‡^ *P* < 0.05 compared with the green group.

Data are expressed as the mean ± standard deviation.

### Conjunctival Goblet Cell Density

**Fig 5:**
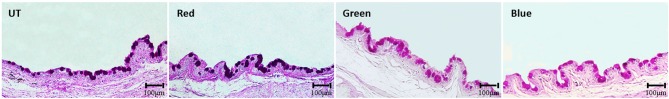
Periodic acid Schiff stains of representative conjunctival specimens in the untouched (UT), red, green, and blue groups at day 10.

The mean density of the conjunctival goblet cells was 16.56 ± 2.06 cells/100 μm, 15.83 ± 2.26 cells/100 μm, 16.11 ± 2.17 cells/100 μm and 15.83 ± 1.98 cells/100 μm in the UT, red, green, and blue groups, respectively. There was no significant difference in goblet cell density between groups (*P* = 0.108, Fig 5).

### Flow Cytometric analysis

**Fig 6:**
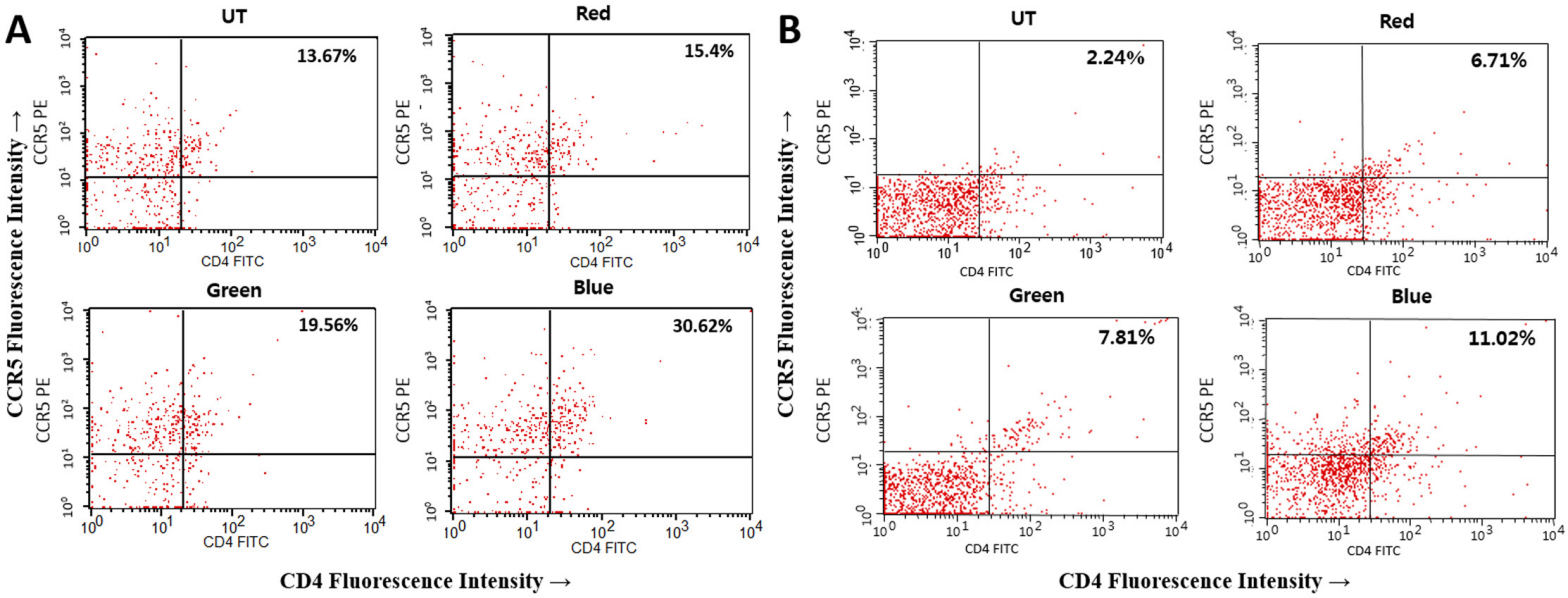
Flow cytometry showing CD4+CCR5+ T cells in the cornea (A) and conjunctiva (B) of the untouched (UT), red, green, and blue groups at day 10.

CD4+CCR5+ T cell percentage histograms from representative corneal and conjunctival samples from UT, red, green, and blue groups are shown in Fig 6A and 6B. In the corneal specimens, the respective percentages of CD4+CCR5+ T cells of UT, red, green, blue groups were 15.22% ± 1.45%, 18.69% ± 2.86%, 20.19% ± 0.55%, and 31.83% ± 1.46%. CD4+CCR5+ T cells significantly increased in the blue group compared to UT, red, and green groups (*P* = 0.001, 0.002, and 0.007, respectively). For the conjunctival specimens, CD4+CCR5+ T cell percentage were 2.70% ± 0.84%, 6.40% ± 1.59%, 6.35% ± 1.27%, and 11.83% ± 1.92% in the UT, red, green, and blue groups, respectively. CD4+CCR5+ T cells significantly increased in blue group compared to the UT group (*P* = 0.023).

### Levels of ROS

ROS levels in the cornea were measured to assess net oxidative stress. A significant increase was observed in the blue group compared to the UT, red and green groups (all *P* < 0.01) (Table 3).

**Table 3:** Corneal reactive oxygen species (ROS) levels in the untouched (UT), red, green, and blue groups.

Group	ROS (DCF fluorescein intensity)
UT	103.46 ± 3.87
Red	107.52 ± 4.52
Green	111.56 ± 3.96
Blue	186.54 ± 5.85*^†^^‡^

* *P* < 0.05 compared with the UT group

^†^ *P* < 0.05 compared with the red group

^‡^ *P* < 0.05 compared with the green group.

Data are expressed as the mean ± standard deviation.

### TUNEL assay

**Fig 7:**
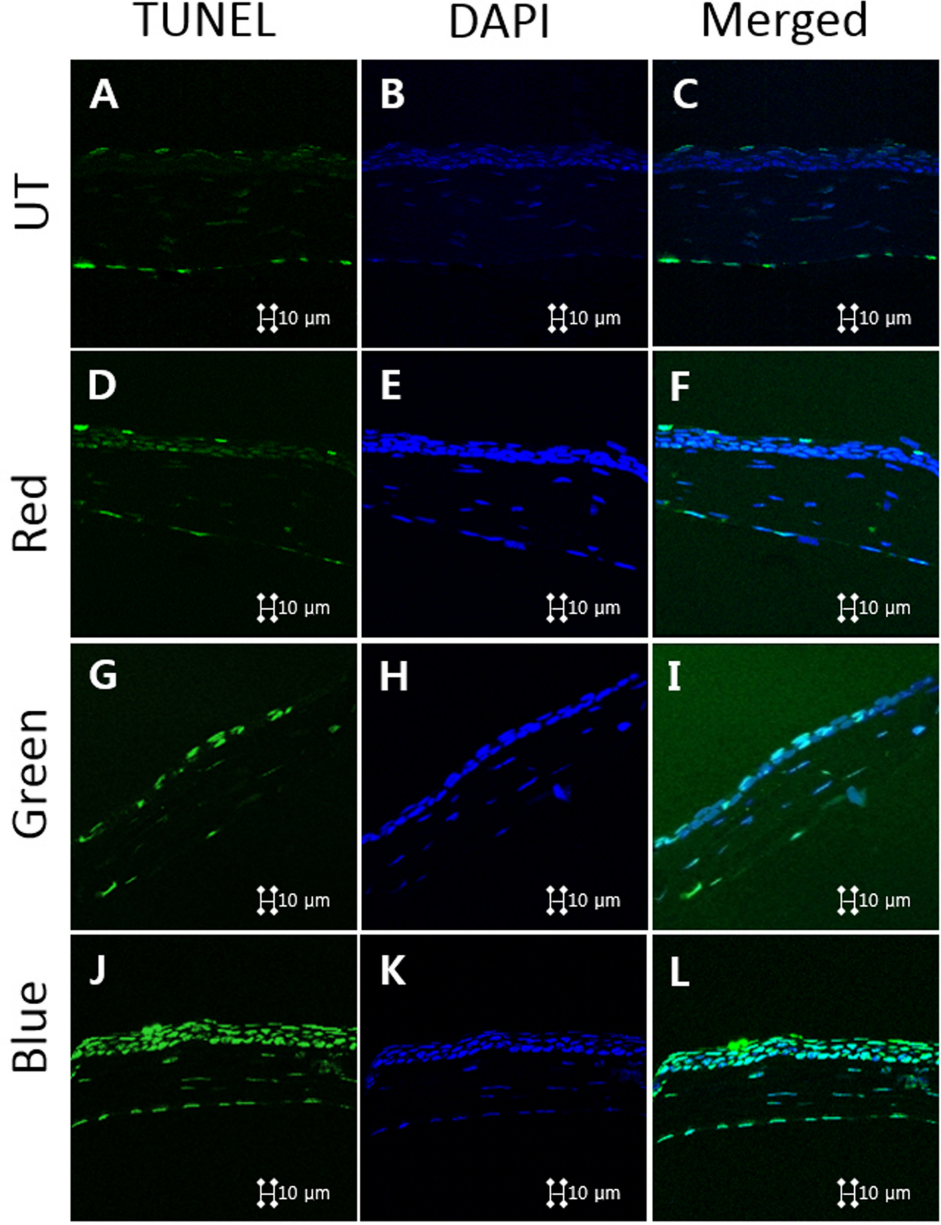
Representative images for Terminal dUTP nick-end labeling (TUNEL) assay showing the apoptotic cells in the cornea of the untouched (A, B, C), red (D, E, F), green (G, H, I), and blue (J, K, L) groups at day 10.

Magnification images of the representative corneal sections stained with TUNEL assay (green fluorescence) and counterstained with DAPI (blue fluorescence) are demonstrated in Fig 7. The blue light irradiation resulted in more apoptotic cells in the superficial corneal epithelium than in the red and green groups. Meanwhile, the red and green groups had some apoptotic cells, but less than the blue group. Additionally, there were hardly any apoptotic cells in the UT group.

## Discussion

The cornea is regularly exposed to sunlight (infrared, visible, and UV light) and atmospheric oxygen. The majority of UV radiation is absorbed by the cornea, suggesting that the cornea may be highly susceptible to the damage caused by UV radiation (especially UVB radiation); the hostile effects of excessive UV radiation to the cornea have been extensively studied [25]. Overexposure to UVA (315 ~ 400 nm) and UVB (280 ~ 315nm) light may cause damage to the corneal epithelium, whereas exposure to UVC radiation (100 ~ 280 nm) can induce deeper lesions in the corneal stroma and Bowman membrane. This can lead to corneal opacity and neovascularization [26]. Those pathological changes caused by UV irradiation are primarily induced by excessive ROS formation.

Oxidative stress induced by UVB in the ocular surface epithelium upregulates the expression of proinflammatory cytokines (e.g., IL-1, IL-6, and IL-8, through the c-jun amino-terminal kinase [JNK] pathway, p38 pathway, and nuclear factor–kB [NF-kB] pathway) and enzymes (e.g. MMP-1) that mediate prostaglandin and leukotriene biosynthesis, as well as antioxidant enzymes in corneal epithelial cells [27–31]. These findings suggest that UV light can induce inflammation and tissue damage. The severity of damage induced by light depends on radiation intensity, radiation wavelength and time of exposure [32,33].

Any form of radiant energy (not limited to UV light) is potentially hazardous if it reaches the eye and is absorbed by the tissue of the eye at levels capable of causing photochemical reactions, heat, structural changes or metabolic disturbance. Infrared ray usually only causes corneal irritation, but high energy (> 30 J/cm^2^) light may also cause deep stromal lesions and even perforations [34]. In the spectrum of visible light, blue light is highly energized and is close in the color spectrum to ultraviolet light, so that overexposure to blue light is potentially hazardous to the eye [35].

The effect of blue light LED on the retina has been extensively studied. Current studies have proven that blue light damage may occur by photosensitizing, oxygen-dependent processes that may affect photoreceptors and RPE through lipid peroxidation and free radical-mediated apoptosis [36]. Indeed, compared to other tissues, the retina is particularly prone to ROS generation due to very high oxygen levels in the choroid, extraordinary high metabolic rates, and exposure to light, particularly light of shorter wavelengths [37].

Among these various wavelengths of visible light, blue light irradiation increased IL-1β, IL-6, and MDA in the corneas, compared to UT, red, and green light irradiation. Both IL-1β and IL-6 are proinflammatory cytokines that are secreted on the ocular surface in response to various pathologic stimuli, such as benzalkonium chloride-induced dry eye, desiccating stress induced dry eye, and microbial infections, such as P. aeruginosa, O. volvulus, or HSV-1 [20,38–40]. IL-6, along with other pro-inflammatory cytokines, plays a major role in the pathogenesis of several inflammatory disease such as dry eye, graft-versus-host disease, and thyroid-associated ophthalmopathy through NF-κB and mitogen-activated protein kinase (MAPK) pathways [41,42]. In addition, previous studies have shown that increased IL-1β induced the loss of corneal epithelial barrier function associated with ocular surface inflammation [43].

Increased inflammatory responses after blue light irradiation was also demonstrated through analysis of CD4+ T cell infiltration with flow cytometry. Homing and infiltrating T cells on the ocular surface in inflammatory conditions, such as dry eye disease, consists predominantly of CD4+ T cells. Th1-related chemokine receptors, such as CXCR3 and CCR5, and their ligands play an important role in the trafficking of activated CD4+ T cells [44]. We have previously found that desiccating stress stimulates the expression of inflammatory cytokines and Th-1 chemokines and their receptors, CCR5 and CXCR3, in the tear film and ocular surface of an experimental dry eye model [18,45].

To evaluate the oxidative stress in the ocular surface after visible light irradiation and apoptotic cell death, we estimated the concentration of the oxidative stress marker MDA, which is a reactive intermediate in the formation of advanced lipoxidation endproducts and known to react with deoxynucleoside to produce various adducts and damage to DNA [46,47]. We detected an increase of MDA in corneas exposed to blue light irradiation compared to their time-matched control. This increased level of MDA means increased lipid peroxidation, which indicates a secondary reaction to ROS formation [48,49]. To measure overall ROS production and cellular apoptosis, we performed a DCF-DA assay and TUNEL stain. Our results demonstrated an increased level of ROS production and apoptosis in the cornea. These findings are consistent with previous reports regarding the relationship between visible light irradiation, cellular ROS levels and corneal epithelial cell viability *in vitro* [16,17].

It is widely recognized that ocular surface inflammation, oxidative stress, and apoptosis play a critical role in the pathogenesis of dry eye [50,51]. Increased pro-inflammatory cytokines and inflammatory cell infiltration are found in the ocular surface of patients with dry eye, and a variety of anti-inflammatory agents have proven effectiveness in decreasing the production of inflammatory cytokines and chemokines, and managing dry eye symptoms and signs [52,53]. Oxidative stress is one of the initiators in activating the signal transduction pathways (e.g. increased MMP-9 and activation of MAPK pathway) implicated in the breakdown of the integrity and inflammatory response of the ocular surface in experimental dry eye [14]. Increased levels of MDA were also found at the ocular surface in patients with dry eye syndrome [25]. In addition, oxidative stress markers increased in the tear film of dry eye patients with Sjögren’s syndrome and were correlated with disease severity [54]. Taken together, these data demonstrate a close relationship between ROS production, lipid peroxidation-related membrane damage, and inflammation in dry eye disease. Therefore, we hypothesized that upregulated oxidative stress, increased inflammatory response and apoptosis could manifest as clinical dry eye in our blue light irradiation model as shown by decreased TBUT and increased corneal staining scores.

The reason why blue light, not visible light with any other wavelength, causes oxidative stress on the cornea is not clear. In previous reports regarding blue LED light irradiation on the retina, short wavelength blue LED light more severely damaged photoreceptor-derived cells than white or longer wavelength green LED light at identical energy levels.^10^ It has been reported that shorter wavelength light increased ROS levels more compared to longer wavelength light exposure [55]. As Grimm et al. [56] postulated, different wavelengths and different intensities of light have specific physical properties that can differentially affect biological molecules, and it is possible that blue light induces oxidative stress more easily than other visible light with different wavelengths. However, any radiant energy above a certain energy level can produce a response after reaching the target tissue and being absorbed, as some experimental setups have used green light to induce retinal damage and illumination with green light in the range of several hours or days eventually led to retinal damage [57]. Another possible explanation is that among spectrums of visible light, lights with shorter wavelengths (e.g., blue light) are absorbed more easily by the cornea than lights with longer wavelength, as shown in a study by Kraats et al. [58]

In our study, an energy level of 50 J/cm^2^ was required to induce significant damage on the corneal surface. This energy level is approximately 20-fold more intense than that used in a previous study regarding UVB radiation and corneal damage [59]. This energy level is approximately 50-fold more intense than that required to induce oxidative damage in the retina [2,60], which is consistent with the previous study [16]. As a first-line biological barrier against irradiant energy, the cornea has been proven to have a powerful antioxidative defense mechanism. In healthy corneas, a number of antioxidant protective mechanisms are present to minimize and reduce damage from ROS [61]. Indeed, 20~40% of the soluble protein content of the cornea is an isoenzyme of aldehyde dehydrogenase, which directly absorbs UV light and removes cytotoxic aldehydes produced by lipid peroxidation [62]. Furthermore, the cornea contains antioxidant enzymes such as superoxide dismutase, catalase, and glutathione peroxidase that scavenge ROS [25]. It may be reasonable to assume that a relatively higher energy level is required to induce oxidative damage in the cornea than in the retina. In addition, the overall absorption rate of visible light into the cornea is about 3%, which is much less than its UV light absorption [58]. Hence, we postulated that the energy level required for blue light to damage the cornea is much higher than the level required for blue light to damage the retina or UV light to damage the cornea.

Significant damage of the conjunctiva after light irradiation was not found in this study. Damage thresholds of the cornea and conjunctiva under UV and visible light spectra did not show much difference, and after irradiation with high energy level visible light (100 J/cm^2^), conjunctival damage manifested as chemosis [26,63]. We hypothesized that among tissues constituting the ocular surface, most of the conjunctiva was covered by mouse eyelid, whereas the cornea itself was solely exposed to the light irradiation, thereby explaining the significant damage only to the cornea. Additionally, we evaluated cellular apoptosis in the meibomian glands and lacrimal glands using TUNEL stain, and results revealed that effect of blue light on the meiboman glands and lacrimal glands is limited, without significant changes in the cellular apoptosis between groups (data not shown).

Taken together, our results indicate that overexposure to visible light with short wavelengths (410 nm) can increase inflammatory markers and oxidative stress, induce apoptosis, and thus may aggravate clinical dry eye parameters in mouse compared with visible light at other wavelengths. Because we evaluated the effect of blue light irradiation using a murine model, responses against blue light irradiation might be variable in clinical situations. Humans are not ordinarily exposed to blue light with high energy intensity, as used in our experiments. However, it is possible that under specific working circumstances, humans can be exposed to high-energy blue light irradiation for a long period of time. In that case, our study can be of help to set up standards about how much and how long human can be exposed to high intensity blue light.

In conclusion, we should at least pay attention to the potential detrimental effects of high-energy blue light irradiation doses on ocular surface health.
