# Correction: Influence of Light Emitting Diode-Derived Blue Light Overexposure on Mouse Ocular Surface

**DOI:** 10.1371/journal.pone.0167671

**Published:** 2016-11-30

**Authors:** Hyo Seok Lee, Lian Cui, Ying Li, Ji Suk Choi, Joo-Hee Choi, Zhengri Li, Ga Eon Kim, Won Choi, Kyung Chul Yoon

The phrase “immunohistochemistry with 4-hydroxynonenal” should be deleted from the last sentence of the abstract. The correct sentence is: Flow cytometry, 2’7’-dichlorofluorescein diacetate (DCF-DA) assay, histologic analysis, and terminal deoxynucleotidyl transferase-mediated dUTP-nick end labeling (TUNEL) staining were also performed.
